# Biosensing Membrane Base on Ferulic Acid and Glucose Oxidase for an Amperometric Glucose Biosensor

**DOI:** 10.3390/molecules26123757

**Published:** 2021-06-20

**Authors:** Gabriela Valdés-Ramírez, Laura Galicia

**Affiliations:** Chemistry Department, Universidad Autónoma Metropolitana Unidad Iztapalapa, Av. San Rafael Atlixco 186, Leyes de Reforma 1ra Secc., 09340 Ciudad de Mexico, Mexico

**Keywords:** ferulic acid, glucose oxidase, electropolymerization, glucose, electrochemical biosensor

## Abstract

A biosensing membrane base on ferulic acid and glucose oxidase is synthesized onto a carbon paste electrode by electropolymerization via cyclic voltammetry in aqueous media at neutral pH at a single step. The developed biosensors exhibit a linear response from 0.082 to 34 mM glucose concentration, with a coefficient of determination R^2^ equal to 0.997. The biosensors display a sensitivity of 1.1 μAmM^−1^ cm^−2^, a detection limit of 0.025 mM, and 0.082 mM as glucose quantification limit. The studies reveal stable, repeatable, and reproducible biosensors response. The results indicate that the novel poly-ferulic acid membrane synthesized by electropolymerization is a promising method for glucose oxidase immobilization towards the development of glucose biosensors. The developed glucose biosensors exhibit a broader linear glucose response than other polymer-based glucose biosensors.

## 1. Introduction

In the past years, glucose biosensors have been extensively used in the alimentary, environmental, and biomedical fields. In the biomedical industry, glucose biosensors are used as an important tool to monitor glucose in biological fluids allowing better management of diabetes, a pathology that is estimated to affect 7.7% of the population by 2030 [1,2,3]. In the food industry, glucose is also a target analyte in the production process [4,5]. Therefore, glucose biosensors development is a major goal of different research groups; the emphasis is on improving the analytical parameters of the existing biosensors, such as linearity covering low and high glucose concentrations and making their fabrication simple, portable, with a rapid and accurate response [1,2,3]. The improvement of analytical parameters can be achieved by modifications of the biosensing membrane synthesis, utilizing modified recognized elements, redox mediators, or nanomaterials, as well as using different immobilization methods by employing a variety of chemicals that allow the synthesis of strong biosensing membranes [1,3]. Knowing that biosensor performance is strongly dependent upon the enzyme immobilization process [6], several research groups have explored enzyme immobilization by electropolymerization utilizing conductive and non-conductive polymers [3,7,8,9,10,11,12,13,14,15]. 

This study presents the development of a glucose biosensor employing ferulic acid as a monomer for the synthesis of the biosensing membrane, with direct incorporation of glucose oxidase (GOx) as the biorecognition element and bovine serum albumin (BSA) as an enzyme stabilizer. The 3-methoxy-4-hydroxycinnamic acid, better known as ferulic acid (FA), is found at the plant cell wall and is the most abundant hydroxycinnamic acid in the plant kingdom [16]. It possesses anti-inflammatory, anticancer, and antioxidant properties; therefore, FA is widely used in the pharmaceutical and food industries [17,18,19]. Likewise, it has been incorporated into biocompatible materials with biomedical applications [20,21]. Due to FA abundance and applications, electrochemical methodologies to detect and quantify FA in real samples have been developed, with studies showing the possibilities of FA electropolymerization, which begins with the monomer oxidation and subsequent ortho-quinone/ortho-hydroquinone system formation, followed by a radical coupling reaction allowing the polymerization [21,22,23,24,25,26]. 

In the present study, the biosensing membranes are synthesized by electropolymerization onto a carbon paste electrode in aqueous media. To the best of our knowledge, this is the first time that ferulic acid has been used as a monomer to synthesize a biosensing membrane incorporating GOx as a biorecognition element to generate amperometric glucose biosensors.

## 2. Results and Discussion

### 2.1. Ferulic Acid Electropolymerization

**Figure 1:**
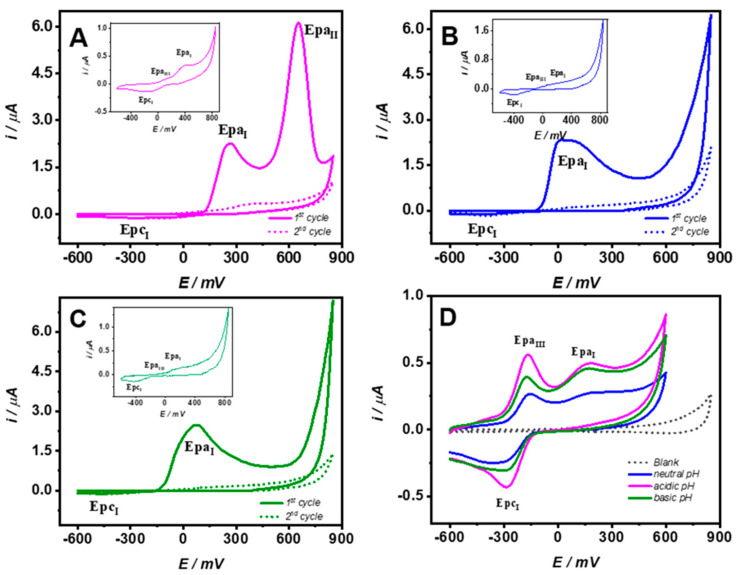
Cyclic voltammograms at the first and second cycles for ferulic acid electropolymerization, 2 mM FA solution prepared at 100 mM acetate at different pH values (**A**) 2.8, (**B**) 6.8, (**C**) 9.6, the insets show a zoom of the second cycle showing the Epa_III_ signal. (**D**) Cyclic voltammogram in PBS at bare CPE and modified CPE with poly-FA synthesized at 2.8, 6.8, or 9.6 pH conditions. All experiments were performed at 20 mV/s.

Poly-ferulic acid membranes were synthesized by electropolymerization via cyclic voltammetry from −600 to 850 mV, 20 mV/s scan rate during 10 cycles onto a CPE in 2.0 mM FA solution prepared in 100 mM acetate medium. Figure 1A–C shows the voltammetric responses for the first and second cycles of FA electropolymerization at three different pH conditions (A) 2.8, (B) 6.8, and (C) 9.6. For the acidic media at the forward scan, two oxidation signals with a maximum current at 260 and 660 mV (Epa_I_ and Epa_II_) were exhibited. Nonetheless, at neutral and basic pH, only one wide signal (Epa_I_) from −80 to 300 mV was displayed. For the backward scans, there was a small reduction signal (Epc_I_) detected at all studied pH levels. At the second and successive cycles, the anodic current decreased rapidly, whereas the reduction currents persisted. The behavior is explained by the electrodeposition of a strongly passivating polymer layer onto the electrode surface; similar behavior was observed for phenols and aromatic amines electropolymerization [3,10,11,12,15,27,28]. Furthermore, at the three systems after the first cycle, a small oxidation at less positive potentials (Epa_III_) appeared (figures inset). Following FA electropolymerization, the modified CPEs were thoroughly rinsed with 100 mM phosphate buffer pH 7.3 (PBS). Figure 1D shows typical voltammograms in PBS, before and after poly-FA electropolymerization, the respective pH modification conditions are shown at the inset legend. For the bare CPE, a flat voltammogram was observed, whereas at modified CPE (poly-FA CPE), two oxidation and one reduction peaks independent of pH were exhibited, Epa_I_ with a maximum current around 180 mV associated to the remaining unattached monomer. Epc_I_ and Epa_III_ were taken as an indication of the presence of poly-FA membrane. Similar results were observed by da Silva [21]. The results showed that under our studied conditions, the electropolymerization of FA can take place at the three pH’s media and not only at aqueous acidic, aqueous basic, or organic media as was found by da Silva and Matsushita in their respective studies [21,22,23]. Considering the biosensor uses underphysiological conditions and to avoid pH changes from the biosensing membrane synthesis to its applications, enzyme immobilization was performed under neutral pH conditions in the presence of acetate.

### 2.2. Ferulic Acid Electropolymerization

**Figure 2:**
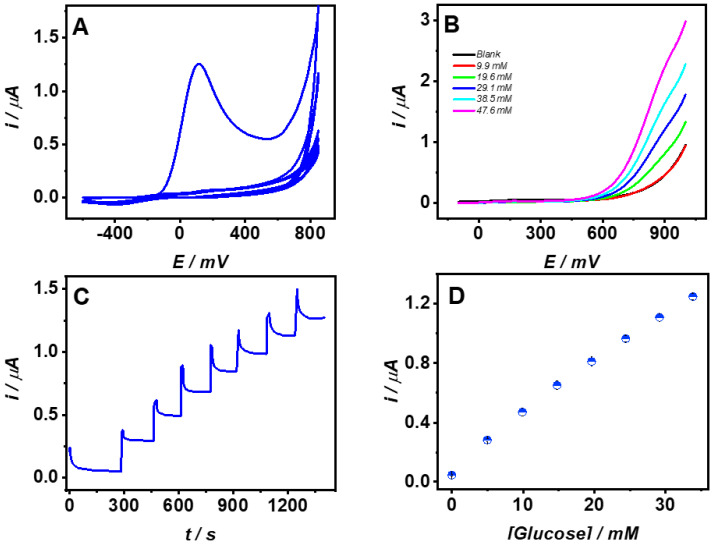
(**A**) Cyclic voltammogram for the synthesis (electropolymerization) of the biosensing membrane of poly–FA–GOx–BSA. (**B**) Linear voltammograms for biosensors at glucose evaluation in PBS. (**C**) Current *vs* time plot at 800 mV for successive glucose 4.9 mM additions (continues monitoring) in PBS. (**D**) Glucose response for three successive calibrations in PBS.

Ferulic acid 2 mM solution prepared in 100 mM acetate pH 7.0, containing 31.5 U/mL GOx and 0.05 mg/mL BSA was employed for biosensing membrane electropolymerization. The biosensing membrane was synthesized by cyclic voltammetry from −600 to 850 mV at 20 mV/s scan rate, during 10 cycles. Figure 2A shows the cyclic voltammogram from FA–GOx–BSA electropolymerization. After the first cycle, a decrease of the anodic peak current was observed; a similar performance was observed at other monomers such as aminophenol and phenylenediamine used for biosensors fabrication by electropolymerization [3,10,11,12,15,27,28].

Following the electropolymerization, the unattached FA, GOx, and BSA were removed by rinsing the biosensors thoroughly with PBS. Glucose biosensors response was examined first by voltammetry. Figure 2B shows the linear sweep voltammetry (LSV) for glucose additions up to 47 mM in PBS at 20 mV/s. The results demonstrate that GOx was incorporated successfully into the poly-FA membrane by the electrochemical detection of the enzymatically produced H_2_O_2_, which can penetrate and diffuse through the biosensing layer, allowing glucose detection. The LSV shows that H_2_O_2_ oxidation began at 600 mV. Therefore, glucose calibrations were performed at 800 mV constant potential in PBS. Figure 2C shows the typical current *vs* time response for successive additions of glucose 4.9 mM. After each glucose addition, the current increased quickly to a steady-state, 90% of the maximum current was reached after 35 s. The current was stable at least for the following 180 s showing good response stability at both low and high glucose concentrations. Figure 2D shows the average results for three consecutive glucose calibrations. The biosensors showed a linear response from 0.082 to 34 mM glucose concentration covering the normal physiological conditions and the hypo and hyperglycemic ranges for human biofluids. The glucose biosensors sensitivity was 1.1 μAmM^−1^ cm^−2^, the coefficient of determination was R^2^ = 0.997, and for three consecutive calibrations, the RSD was 1.57% showing acceptable operational stability. LOQ and LOD were 0.082 and 0.025 mM calculated as $10\;\sigma_{B}/b\;and\;3\sigma_{B}/b$, respectively [29]. The results demonstrate that the immobilization method employing FA as a monomer could be suitable for future applications on continuous glucose monitoring. Furthermore, the biosensors long-term operational stability was determined by performing daily glucose calibration in PBS. After 7 days, the maintained sensitivity was 64.3% of the initial amperometric response. The sensors were stored at room temperature in dry conditions while not in use. Similar synthesis conditions were employed to prepare and evaluate an independent set of five different biosensors; the coefficient of variation for the sensitivity of the biosensors at first glucose calibration was 3.6%. The biosensors’ selectivity was evaluated for two electroactive species present in human plasma under normal physiological conditions, 300 μM of uric acid (UA) and 100 μM of ascorbic acid (AA) at polyFA membrane by amperometry at 800 mV in PBS. It was found that only 10 and 15% of the responses for UA and AA were generated compared to bare CPE. The signal is attributed to the high potential applied for the electrochemical detection. Yet, the poly-FA membrane synthesized at neutral pH could work as a permselective membrane; in this regard, a total rejection for AA and UA applying lower detection potential at poly-FA membranes synthesized at acidic conditions onto multi-wall carbon nanotubes were found [21]. 

To evaluate the performance of the biosensor in the presence of proteins, the synthesized biosensors were used to perform glucose calibration in artificial interstitial fluid (a-ISF) prepared according to Fogh-Andersen [30]; CO_2_ was not added into the solution. The results showed that biosensor sensitivity in a-ISF decreased 20%; nonetheless, the linearity was maintained up to 30 mM glucose concentration, the sensitivity diminution could be associated with non-specific BSA adsorption. After glucose calibrations in a-ISF, the biosensors were washed and re-calibrated in PBS; 99.8% of the original sensitivity prevailed.

The analytical parameters of the developed glucose biosensors based on poly–FA–GOx–BSA membrane show a competitive performance compared to other polymer-based biosensors. The biosensing membrane synthesized in this work excelled in the linear range. A comparison of the analytical parameters for the developed glucose biosensor and other polymer-based glucose biosensors is shown at Table 1. 

Usually, the polymer-based biosensors and others are saturated around 10 mM glucose concentration. Thus, their use in samples where the glucose concentration is larger, such as samples from diabetic patients where glucose concentrations in the hyperglycemic conditions could be larger than 22 mM glucose [31], and the use of other biosensing membranes is useless. In this regard, the developed biosensor in this work did not show saturation even at 34 mM glucose concentration, making the use of the developed biosensing membrane attractive in improving the linear range of glucose biosensors employed in the biomedical field, either as strip sensors or continuous glucose monitoring sensors (CGM). 

## 3. Materials and Methods

Ferulic acid, glucose oxidase, bovine serum albumin, glucose, ascorbic acid, and uric acid were purchase from Sigma-Aldrich; sodium acetate three hydrated, sodium chloride, potassium phosphate monobasic, potassium phosphate dibasic, and sodium chloride were purchase from J.T. Baker; graphite powder 99.99% purity was obtained from Alfa Aesar; and mineral oil was purchased from Fluka. 

All chemicals were used as received without further purification. Ferulic, uric, and ascorbic acid solutions were daily prepared with Milli-Q water 18.2 MΩ and stored in the dark until used. To reach an equilibrium between glucose isomers, glucose stock solutions were prepared at least 48 h before use. Voltammetric and amperometric experiments were carried out at room temperature. The electrochemical working station was a BAS100 potentiostat employing a three-electrode system, where a platinum wire was used as a counter electrode, saturated mercury sulfate (Hg/Hg_2_SO_4sat_) was used as a reference electrode, and carbon paste electrode (CPE) or modified CPE was used as a working electrode.

Working electrodes were prepared from a homogeneous paste 1:1 mixture of graphite powder and mineral oil, the paste was firmly packed into a PVC tube that provided a geometrical surface area of 0.0314 cm^2^. The electrical contact was copper wire. After the carbon paste was packed and before modification, the electrode surface was smoothed utilizing white, bond paper. CPE was modified either by the electropolymerization of FA or FA–GOx–BSA. At first, the electropolymerization of ferulic acid was carried out at different pH conditions in acetate medium. The presence of poly-FA membrane on a CPE was evaluated by cyclic voltammetry. The biosensing membranes were synthesized in acetates medium at neutral pH in a solution containing a monomer (FA), a biorecognition element (GOx), and an enzyme stabilizer (BSA). The membrane synthesis was performed by cyclic voltammetry at a scan rate of 20 mV/s, from −600 to 850 mV for 10 cycles.

To obtain the biosensor analytical parameters, the electrodes were evaluated through their response to glucose performing calibration. The glucose quantification was performed at constant potential by amperometry through successive glucose additions. Biosensor selectivity was evaluated for ascorbic acid as well as uric acid responses. First, the glucose biosensors were evaluated in PBS, and later, in the presence of proteins utilizing artificial interstitial fluid.

## 4. Conclusions

Concerning ferulic acid electropolymerization, the results obtained in this investigation have shown the feasibility of a poly-FA membrane synthesis in aqueous media at acidic, at basic, and also at neutral pH, increasing the potential use of the polymeric membrane for the development of biosensing films.

We effectively synthesized a poly-FA–GOx–BSA biosensing membrane toward the development of amperometric glucose biosensors. The developed glucose biosensors exhibited a linear response in a dynamic range covering the hypo, normal, and hyperglycemic conditions showing favorable results concerning linearity improvement of glucose biosensors. Moreover, the biosensors showed a rapid response as well as good operational stability. Further, the results obtained on a-ISF were encouraging for biosensors used at real samples (biofluids). The versatility of the system would allow its implementation for other recognition elements in the direction of biosensors with applications in healthcare, the food industry, or environmental fields. Future work will be focused on improving analytical parameters employing other electrode materials, redox mediators, or permselective membranes to improve selectivity, stability, sensitivity, repeatability, shelf life, and linearity to be used on food samples without previous treatment. Morphological and structural biosensing membrane characterization studies will also be performed in future work. 

## Figures and Tables

**Table 1:** Comparison of polymer-based glucose amperometric biosensors.

Material	Detection Limit	Linear Range	Sensitivity	Ref.
PANI/GOx	0.062 mM	1–10 mM	0.54 μA mM^−1^	[8]
Nanodiamond-g-PANI/GOx	0.018 mM	1–30 mM	2.03 μA mM^−1^	[8]
PANI/MWCNTs/GOx/HRP	0.02 mM	0.5–12 mM	0.94 μA mM^−1^	[9]
Pt/o-PD/βcyclodextrin/GOx	0.79 mM	2.5–15.5 mM	0.150 nA mM^−1^	[10]
Ag/p(m-aminophenol) nanofibers	0.062 μM	0.1–8.0 mM	17.45μA mM^−1^ cm^−2^	[11]
Pt/poly(o-PD)/GOx	0.020 mM	0–2 mM	0.140 nA mM^−1^	[12]
Polypyrrole/CNT/GOx	0.005 mM	1–4.1 mM	54.2 μA mM^−1^ cm^−2^	[13]
Pt/poly(o-PD)/GOx	0.100 mM	0–14 mM	353 μA mM^−1^	[15]
CPE/poly-FA–GOx–BSA	0.025 mM	0.082–34 mM	1.1 μAmM^−1^ cm^−2^	This work

## Data Availability

Not applicable.
